# Expression of human endogenous retroviruses (HERVs) in the blood of adolescents with and without obesity

**DOI:** 10.1038/s41366-026-02129-9

**Published:** 2026-06-29

**Authors:** Massimiliano Bergallo, Ivana Rabbone, Cristina Calvi, Anna Pau, Cristina Partenope, Anna Clemente, Simonetta Bellone, Maddalena Dini, Ilaria Galliano, Pier-Angelo Tovo

**Affiliations:** 1 University of Turin, Department of Public Health and Pediatric Sciences, Medical School, Turin, Italy; 2 Laboratory of Specialistic Pediatry, Department of Children’s Pathology and Care, Regina Margherita Children’s Hospital, Turin, Italy; 3 Division of Paediatrics, Department of Health Sciences, University of Piemonte Orientale, Novara, Italy

**Keywords:** Nutrition disorders, Medical research

## Abstract

**Background/Objectives:**

Human endogenous retroviruses (HERVs) are genomic elements derived from ancient retroviral infections and are increasingly recognized for their roles in gene regulation and immune modulation. Obesity is characterized by chronic low-grade inflammation and metabolic dysregulation, which may influence HERV expression.

**Subjects/Methods:**

In this study, we evaluated the transcriptional levels of HERV-H-*pol*, HERV-K-*pol*, and HERV-W-*pol* in peripheral blood from adolescents with obesity as compared to healthy normal weight controls (HC). Total RNA was extracted from whole blood samples, and gene expression was assessed using quantitative reverse-transcription PCR (RT-qPCR), with GAPDH as a reference gene.

**Results:**

HERV-H-*pol* and HERV-K-*pol* were significantly upregulated in adolescents with obesity as compared to HC, whereas HERV-W-*pol* was downregulated. No significant associations were observed between HERV expression levels and age, sex, or metabolic parameters.

**Conclusions:**

These findings suggest that obesity in adolescents is associated with a differential regulation of HERV elements, possibly reflecting the impact of chronic inflammatory and metabolic stress. HERV dysregulation may represent a novel molecular feature of obesity and warrants further investigation as a potential biomarker or contributor to obesity-related complications.

**Figure:**
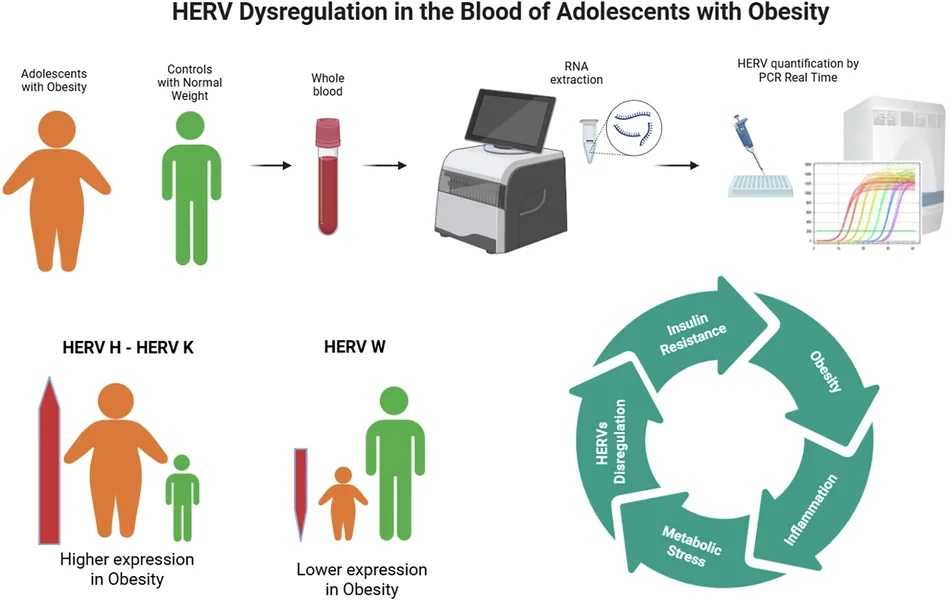

## Introduction

Childhood obesity is a major global health concern, associated with metabolic complications, such as insulin resistance and a state of chronic low-grade systemic inflammation [1, 2]. These alterations are linked to complex transcriptional and epigenetic changes that may contribute to disease progression [3].

Human endogenous retroviruses (HERVs) are genomic elements derived from ancient retroviral infections, accounting for approximately 8% of the human genome [4]. Once considered non-functional, they are now recognized as regulators of gene expression and immune responses, and their dysregulation has been implicated in inflammatory and metabolic disorders [5–7].

HERV expression is influenced by epigenetic mechanisms and inflammatory signaling pathways, both of which are altered in obesity [3, 5, 8, 9]. While HERV activation has been described in several pathological conditions, including metabolic diseases in adults, its role in pediatric obesity remains largely unexplored.

In this study, we evaluated the expression levels of the *pol* genes of HERV-H, HERV-K, and HERV-W in peripheral blood from adolescents with obesity, compared to healthy controls, to investigate whether obesity is associated with altered HERV transcription.

## Materials and methods

Adolescents with obesity were recruited at the Department of Pediatrics, Azienda Ospedaliera-Universitaria Maggiore della Carità, Novara, Italy. Obesity was diagnosed as Body Mass Index (BMI) ≥97th percentile for age and sex according to WHO reference standards [1].

The control group consisted of healthy normal-weight adolescents undergoing routine laboratory evaluations, with results within the normal range. Subjects with genetic syndromes, endocrine disorders, or acute illnesses were excluded.

Written informed consent was obtained from the parents or legal guardians. The study was approved by the local Ethics Committee (code 0003495, 17/12/2021) and conducted in accordance with the Declaration of Helsinki.

Peripheral blood samples were collected during routine clinical evaluations. Whole blood (200 μL) was mixed with RNA stabilization solution and stored at −80 °C until processing.

Total RNA was extracted using an automated system (Maxwell® 16, Promega Corporation, Madison, WI, USA) and RNA concentration and quality were assessed by spectrophotometry (NanoDrop 2000™, Thermo Fisher Scientific, Waltham, USA).

cDNA was synthesized from total RNA and gene expression was quantified by quantitative reverse-transcription PCR (RT-qPCR) using specific primer/probe sets for HERV-H-*pol*, HERV-K-*pol*, HERV-W-*pol* and GAPDH as reference gene. Each sample was analyzed in triplicate, and relative expression levels were calculated using the 2^−ΔΔCt^ method [7] and the results were reported as relative quantification (RQ) in arbitrary units.

Statistical analyses were performed using GraphPad Prism (version 7). Differences between groups were assessed using Mann-Whitney test or Fisher’s exact test, as appropriate. Correlations were evaluated with Spearman’s rank test. A *p*-value < 0.05 was considered statistically significant.

## Results

Thirty adolescents with obesity and 35 healthy controls (HC) were included in the study.

Age and sex distribution were comparable between groups: median age was 14.0 years (IQR 12.0–15.1) in adolescents with obesity and 13.8 years (IQR 12.6–15.1) in healthy controls (*p* = 0.7442); the male/female distribution was 17/13 and 16/19, respectively (*p* = 0.4586).

Clinical and metabolic characteristics of adolescents with obesity are summarized in Table 1.

**Table 1:** Clinical and metabolic characteristics of adolescents with obesity.

Variable	Adolescents with obesity (*n* = 30)
Age (years): median (IQR)	14.0 (12.0–15.1)
Sex: M/F	17/13
BMI (kg/m²): median (IQR)	32.5 (28–36.6)
BMI z-score: median (IQR)	2.5 (1.9–2.9)
Glycemia (mg/dL): median (IQR)	85 (80–88)
Insulin (mU/mL): median (IQR)	19.8 (13.1–30.3)
HOMA-IR: median (IQR)	4.1 (2.9–5.4)
Uric acid (mg/dL): median (IQR)	5.6 (4.6–6.1)

*n* number, *IQR* interquartile range (25–75%), *BMI* body mass index, *HOMA-IR* homeostatic model assessment of insulin resistance.

GAPDH cycle threshold (Ct) values were comparable between controls and adolescents with obesity, confirming comparable RNA quality across samples. Median Ct values (IQR 25–75%) were 22.31 (21.79–22.55) in controls and 22.47 (22.15–22.88) in adolescents with obesity (*p* = 0.1016).

**Fig. 1:**
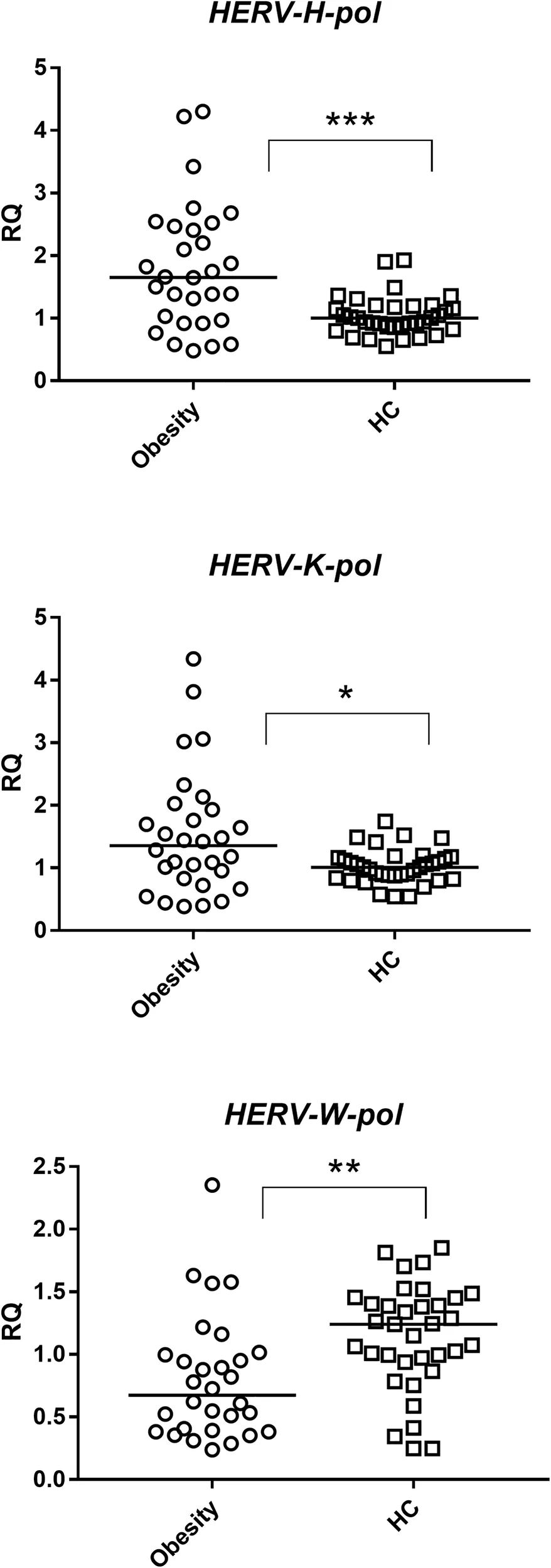
Differential expression of HERV-H, HERV-K, and HERV-W *pol* genes in adolescents with obesity and healthy controls. HERV-H-*pol* and HERV-K-*pol* expression levels were significantly higher, whereas HERV-W-*pol* levels were significantly lower in adolescents with obesity (Obesity) compared with healthy controls (HC). Circles and squares indicate individual biological samples; horizontal lines represent medians. Statistical significance between groups was assessed using the Mann–Whitney test.

HERV-H-*pol* and HERV-K-*pol* expression levels were significantly increased in adolescents with obesity as compared to HC (*p* = 0.0006 and *p* = 0.0373, respectively), whereas HERV-W-*pol* expression was significantly decreased (*p* = 0.0015) (Fig. 1). Median (IQR) values were: HERV-H-*pol*: 1.65 (0.98–2.45) vs. 1.00 (0.86–1.19); HERV-K-*pol*: 1.36 (0.86–1.89) vs. 1.01 (0.86–1.16); HERV-W-*pol*: 0.67 (0.40–0.99) vs. 1.24 (0.95–1.43).

No significant correlations were observed between HERV expression levels and age or sex in either group (Supplementary Figs. S1 and S2). Similarly, no significant associations were found between HERV transcription levels and metabolic parameters, although a trend toward a positive correlation with insulin levels was observed (Supplementary Figs. S3–S5).

## Discussion

In this study, we tested the hypothesis that HERV expression patterns differ between adolescents with obesity and healthy controls. Our findings confirmed this hypothesis, showing that childhood obesity is associated with altered expression of retroviral sequences, with significant upregulation of HERV-H-*pol* and HERV-K-*pol* and downregulation of HERV-W-*pol*. The difference was more marked for HERV-H-pol and HERV-W-pol, whereas the increase in HERV-K-pol, although statistically significant, was less pronounced. The biological mechanisms underlying these findings remain unclear. HERV expression is known to be modulated by epigenetic mechanisms and inflammatory signaling pathways [3, 5, 8]. In obesity, adipose tissue acts as an active endocrine organ, releasing pro-inflammatory cytokines that may influence gene transcription and contribute to HERV activation [10, 11]. In this context, the differential expression observed in our study may reflect the impact of chronic metabolic stress and low-grade inflammation on HERV regulation.

The discordant expression of different HERV families observed in our study is of interest and has been reported in other pathological conditions [12]. HERV-H has been implicated in transcriptional regulation and cellular plasticity, and its upregulation may reflect changes in immune cell function or epigenetic regulation [13]. HERV-K, one of the most biologically active families, has been associated with inflammatory responses [6, 14].

Conversely, the downregulation of HERV-W-*pol* may reflect selective epigenetic silencing or compensatory regulatory mechanisms in response to chronic inflammation [5]. We did not observe significant correlations between HERV expression levels and age, sex or metabolic parameters. This suggests that HERV dysregulation may represent a general feature of obesity rather than being directly associated with specific clinical variables. However, the absence of significant correlations may also be related to the relatively small sample size and the cross-sectional design of the study.

This study has several limitations. The relatively small sample size may limit the statistical power of the analyses, particularly for correlation studies. In addition, the cross-sectional design does not allow conclusions about causal relationships. Finally, we did not assess HERV protein expression or immune responses, which could provide further insight into the functional significance of the observed transcriptional changes. Despite these limitations, our findings provide novel evidence of HERV dysregulation in pediatric obesity, a field that remains largely unexplored. Further studies are needed to clarify the mechanisms underlying these alterations and to evaluate their potential role in obesity-related complications.

## Supplementary information

Supplementary Materilas

## Data Availability

The data supporting this study are available at an aggregate or population level upon reasonable request to the corresponding author.

## References

[1] World Health Organization. Obesity and overweight: Key facts. 2023. https://www.who.int/news-room/fact-sheets/detail/obesity-and-overweight. Accessed 25 May 2025.

[2] Ferrante AW. Obesity-induced inflammation: a metabolic dialogue in the language of inflammation. J Intern Med. 2007;262:408–14. 10.1111/j.1365-2796.2007.01852.x. 17875176

[3] Ling C, Ronn T. Epigenetics in human obesity and type 2 diabetes. Cell Metab. 2019;29:1028–44. 10.1016/j.cmet.2019.03.009. PMC6509280 30982733

[4] Lander ES, Linton LM, Birren B, Nusbaum C, Zody MC, Baldwin J, et al. Initial sequencing and analysis of the human genome. Nature. 2001;409:860–921. 10.1038/35057062. 11237011

[5] Hurst TP, Magiorkinis G. Epigenetic control of human endogenous retrovirus expression: focus on regulation of long-terminal repeats (LTRs). Viruses. 2017;9:130. 10.3390/v9060130. PMC5490807 28561791

[6] Rangel SC, da Silva MD, da Silva AL, dos Santos JMB, Neves LM, Pedrosa A, et al. Human endogenous retroviruses and the inflammatory response: a vicious circle associated with health and illness. Front Immunol. 2022;13:1057791. 10.3389/fimmu.2022.1057791. PMC9744114 36518758

[7] Chuong EB, Elde NC, Feschotte C. Regulatory activities of transposable elements: From conflicts to benefits. Nat Rev Genet. 2017;18:71–86. 10.1038/nrg.2016.139. PMC5498291 27867194

[8] Ibrahim HIM. Epigenetic regulation of obesity-associated type 2 diabetes. Medicina. 2022;58:1366. 10.3390/medicina58101366. PMC9607337 36295527

[9] Livak KJ, Schmittgen TD. Analysis of relative gene expression data using real-time quantitative PCR and the 2^−ΔΔCT^ method. Methods. 2001;25:402–8. 10.1006/meth.2001.1262. 11846609

[10] Zatterale F, Longo M, Naderi J, Raciti GA, Desiderio A, Miele C, et al. Chronic adipose tissue inflammation linking obesity to insulin resistance and type 2 diabetes. Front Physiol. 2020;10:1607. 10.3389/fphys.2019.01607. PMC7000657 32063863

[11] Tourniaire F, Romier-Crouzet B, Lee JH, Marcotorchino J, Gouranton E, Salles J, et al. Chemokine expression in inflamed adipose tissue is mainly mediated by NF-κB. PLoS One. 2013;8:e66515. 10.1371/journal.pone.0066515. PMC3688928 23824685

[12] Lee YJ, Jeong BH, Park JB, Kwon HJ, Kim YS, Kwak IS. The prevalence of human endogenous retroviruses in the plasma of major burn patients. Burns. 2013;39:1200–5. 10.1016/j.burns.2012.12.013. 23339865

[13] Markopoulos GS. A systems biology approach on the regulatory footprint of human endogenous retroviruses (HERVs). Diseases. 2022;10:98. 10.3390/diseases10040098. PMC9680359 36412592

[14] Tugnet N, Rylance PB, Roden D, Trela M, Nelson PN. Human endogenous retroviruses (HERVs) and autoimmune rheumatic disease: is there a link? Open Rheumatol J. 2013;7:13–21. 10.2174/1874312901307010013. PMC3636489 23750183

