# Supplementary material for: Expression of human endogenous retroviruses (HERVs) in the blood of adolescents with and without obesity
Source: Int J Obes (Lond). 2026 Jun 29;50(9):1995–8. doi: 10.1038/s41366-026-02129-9 (PMC13649576; doi:10.1038/s41366-026-02129-9)
Supplement: Supplementary file 1 [file 41366_2026_2129_MOESM1_ESM.docx]

**Supplementary Materials**

**Figure S1.**

Association between HERV expression and age

Scatter plots showing the relationship between HERV-H-*pol*, HERV-K-*pol*, and HERV-W-*pol* expression levels and age in adolescents with obesity (Obesity) and healthy controls (HC). No significant correlations were found in either group (*p* > 0.05). Circles indicate individual biological samples; lines represent linear regression. Statistical analysis: Spearman’s rank test.

**Figure S2.**

HERV expression according to sex

Box plots showing HERV-H-*pol*, HERV-K-*pol*, and HERV-W-*pol* expression levels in males and females in adolescents with obesity (Obesity) and healthy controls (HC). No significant sex-related differences were observed (*p* > 0.05). Circles and squares indicate individual biological samples; horizontal lines represent medians. Statistical analysis: Mann–Whitney test.

**Figure S3.**

Association between HERV expression and body mass index (BMI)

Scatter plots showing correlations between HERV-H-*pol*, HERV-K-*pol*, and HERV-W-*pol* expression levels and BMI values in adolescents with obesity. No significant correlations were observed (*p* > 0.05). Circles indicate individual biological samples; lines represent linear regression. Statistical analysis was performed using Spearman’s rank test.

**Figure S4.**

Association between HERV expression and insulin or HOMA-IR

Scatter plots showing correlations between HERV-H-*pol*, HERV-K-*pol*, and HERV-W-*pol* expression levels and insulin levels or HOMA-IR in adolescents with obesity. No significant correlations were observed (*p* > 0.05), although a trend toward a positive association with insulin levels was noted. Circles indicate individual biological samples; lines represent linear regression. Statistical analysis was performed using Spearman’s rank test.

**Figure S5.**

Association between HERV expression and metabolic parameters

Scatter plots showing correlations between HERV-H-*pol*, HERV-K-*pol*, and HERV-W-*pol* expression levels and metabolic parameters (uric acid and glycemia) in adolescents with obesity. No significant correlations were observed (*p* > 0.05). Circles indicate individual biological samples; lines represent linear regression. Statistical analysis was performed using Spearman’s rank test.
